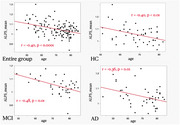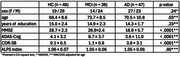# Evaluation of the ALPS Index as a Glymphatic System Biomarker Across the Alzheimer's Disease Continuum

**DOI:** 10.1002/alz70856_102142

**Published:** 2025-12-24

**Authors:** Tony Kaku, Shogyoku Bun, Koki Takahashi, Yu Mimura, Toshiki Tezuka, Masahito Kubota, Yuki Momota, Takayuki Hoshino, Hajime Tabuchi, Keisuke Takahata, Shinichiro Nakajima, Hiroyuki Uchida, Masaru Mimura, Daisuke Ito

**Affiliations:** ^1^ Keio University School of Medicine, Tokyo, Japan; ^2^ National Institutes for Quantum Science and Technology, Chiba, Japan

## Abstract

**Background:**

The glymphatic system has been implicated in the pathophysiology of Alzheimer's disease (AD). The diffusion tensor image analysis along the perivascular space (DTI‐ALPS) method has recently gained attention for assessing the human glymphatic system. We investigated whether the ALPS index differs across disease stages in the AD continuum.

**Method:**

We conducted a cross‐sectional study at Keio University Hospital in Tokyo, Japan (#N20170237). Participants in the AD continuum were categorized into three groups: healthy controls (HC), those with mild cognitive impairment due to AD (MCI‐AD), and those with dementia due to AD (D‐AD). Participants with MCI‐AD and D‐AD were included only if they tested positive for amyloid beta using 18F‐florbetaben positron emission tomography (FBB‐PET). DTI data (b=1000 m/s2, 30 directions) were acquired with 3T magnetic resonance imaging. The ALPS index was calculated from the diffusion tensor of bilateral association and projection fibers. First, we examined the correlation between the ALPS index and clinico‐demographic characteristics. Second, we performed an analysis of covariance (ANCOVA) to compare the ALPS index among these groups, adjusting for the significant correlates.

**Result:**

The study cohort consisted of 48 HC, 38 participants with MCI‐AD, and 47 participants with D‐AD (Table 1). A negative correlation between the ALPS index and age was observed in the combined sample (r=‐0.40, *p* <0.01), as well as within each group (Figure 1). The ANCOVA analysis adjusting for age revealed no significant group difference in the ALPS index (*p* = 0.80, Table 1).

**Conclusion:**

A moderate negative correlation between age and the ALPS index supports age‐dependent dysfunction in the glymphatic system. Unlike previous studies, our analysis revealed no significant differences in the ALPS index across the AD continuum. One potential explanation is that the participants in this study had relatively preserved cognitive function (Table 1) and were at early stages of AD pathophysiology compared with those in previous studies. These early stages may have limited the ability of the DTI‐ALPS method to detect the glymphatic dysfunction. Further research is warranted to evaluate the applicability of the DTI‐ALPS method in detecting early‐stage glymphatic dysfunction within the AD continuum.